# A Comparative Study of Multi-Scale Hybrid Deep Learning Frameworks for Estimation of Domestic Load Demand of Pakistan’s Central Region

**DOI:** 10.3390/s26154991

**Published:** 2026-08-06

**Authors:** Muhammad Yousouf Bashir, Mustafa Shakir, Ali Raza, Manzoor Ellahi, Mohsin Jamil

**Affiliations:** 1 Department of Electrical Engineering, Superior University, Lahore 54000, Pakistan; yousafdhms@gmail.com (M.Y.B.); mustafa.shakir@superior.edu.pk (M.S.); manzoor.ellahi@superior.edu.pk (M.E.); 2 Department of Electrical Engineering, University of Engineering & Technology, Lahore 54000, Pakistan; 3 Department of Engineering, Faculty of Mathematics and Science, Brock University, Saint Catharines, ON L2S 3A1, Canada; mjamil@brocku.ca

**Keywords:** load forecasting, long short-term memory (LSTM), continuous wavelet transform (CWT), SARIMAX, gradient boosting, exogenous calendar variables, stratified error analysis, architecture ablation, multi-lead forecast evaluation, renewable energy resources (RERs), LESCO

## Abstract

In this technological era, electrical energy is the bloodstream for the economic and social development of any country. It is the need of the time that developing countries like Pakistan have strategic planning for efficient generation and utilisation of electricity and have as much cheap electricity as possible at their disposal while respecting environmental constraints. The overloaded and ageing infrastructure of an electrical power network can impact system reliability and the sustainability of power generation, transmission and distribution mechanisms. The initiation of the planning process depends upon accurate load estimation to optimally fulfil consumers’ power needs. This paper compares statistical, hybrid and deep learning (DL) mechanisms, including SARIMAX, SARIMA with gradient boosting (SARIMA-GB), long short-term memory (LSTM) network, STL decomposition with LSTM, and CWT-LeNet-5-LSTM, for the prediction of residential electricity demand in the LESCO region of central Pakistan. The study uses 7670 daily feeder observations recorded between 1 January 2002 and 31 December 2022. The series is modelled at its native daily resolution and partitioned chronologically into a fitting span of 5216 days, a validation span of 920 days and a test span of 1534 days beginning 20 October 2018. All models receive the same block of 14 exogenous calendar variables, four annual Fourier harmonic pairs, day-of-week and month sine and cosine terms, a weekend indicator and a linear trend, and all neural models are trained with a validation split, early stopping and restoration of the best weights rather than for a fixed number of epochs. Accuracy is assessed with MAE, RMSE, MAPE and peak normalized RMSE under one recursive protocol at forecast leads of 1, 7, 14 and 30 days, because the ranking of the frameworks depends on the lead. Averaged over three random initialisations, the proposed CWT-LeNet-5-LSTM attains the lowest error at every multi-step lead, reaching an MAPE of 2.35 ± 0.28% at lead 30 against 3.32% for the multivariate LSTM, 3.89% for SARIMA-GB and 4.47% for SARIMAX. At lead 1, SARIMA-GB is the more accurate model (0.76% against 1.20 ± 0.17%) because the previous day’s observed load dominates one-step prediction for a series whose lag-one autocorrelation is 0.978. An architecture ablation isolates the contribution of the wavelet stage, the convolutional stage, the anisotropic pooling and the calendar fusion. A stratified analysis across seasons, weekdays, weekends and high-, medium- and low-load days shows where the advantage is concentrated. Additionally, Diebold–Mariano tests identify the statistical significance of the differences.

## 1. Introduction

Traditional Grids (TGs) have conventionally been used to generate, transmit and distribute electric power among various categories of consumers. The initially installed infrastructure of TGs was inefficient, resulting in energy wastage through power loss caused by transmission and distribution infrastructure [1]. Moreover, the ageing of energy infrastructure has resulted in reduced efficiency, a drop in reliability and abridged power quality. These issues have caused an increase in fault ratios, frequent load-shedding and major blackouts, and an upsurge in power losses as well [2]. These shortcomings have laid the path for the development of the Smart Grid (SG), which includes both utility and user communication. These modern networks also require the forecasting of power demand and resource availability to ensure the efficient operation of the system [3]. Power firms can better plan for optimal operation of power grids through the aid of precise and efficient forecasting techniques. Forecasting is also carried out to support power suppliers by optimizing power usage and by helping in load shifting from peak to off-peak hours through differentiated tariffs. The forecasting of electrical load helps to maintain a steady flow of power while keeping a check on the over- or under-production of RERs (such as rooftop solar PV systems) included in the power network [4,5].

The continuously growing dependence on electric power (with thermal resources being the major contributor) is resulting in an energy crisis, especially in developing countries, thus impacting system reliability as well [6]. In Pakistan, frequent blackouts have occurred during the last two decades, which have greatly hampered the country’s development. The most recent blackout occurred on 23 January 2023, the second major grid breakdown in the country in two and a half years [7]. It is the need of the time to make strategic plans to cope with the rapidly increasing demand for electric power by incorporating advanced technologies for power generation, transmission, distribution and consumption.

The conventional resources of power generation (coal, oil, gas, etc.) pollute the atmosphere and are depleting rapidly. These factors emphasise shifting toward unconventional resources (solar, wind, tidal, biofuels, etc.) for power generation while considering environmental constraints. Modern grid operations are becoming more complex due to the integration of renewables in power networks [8,9,10]. In this technological era, it is possible for consumers to both use electricity supplied by a utility company and sell electric power back to a utility grid. On this basis, at the load side, consumers are divided into two major categories: consumers and prosumers [11].

Accurate prediction of electrical load is an important consideration to ensure reliable, economically viable and safe operation of a power network [12]. Load forecasting models are categorized as ultra-short, short, medium and long-term procedures according to horizons ranging from hours to years, respectively [13,14]. The statistical enigma of short-term forecasting of electric demand considers weekly to monthly periods, weather impact, holidays, and the economic and financial conditions of the country [15,16,17].

From an economic, reliability and environmental perspective, load forecasting has the following benefits [18,19]:

- It supports reasoned projections for future inspection and power system management;
- It minimizes the risk of damage to power systems by lowering power quality issues;
- It gives a clear idea of areas of investment and possible losses;
- It enables planning for maintenance of systems to avoid major damage to power equipment;
- It helps in reducing the use of fossil fuels and carbon emissions and optimally directs future financial investments.

### 1.1. Research Contribution

In the present paper, the demand of domestic consumers served by LESCO is considered, with the target being the aggregate residential feeder load. Because the feeder may include customers with rooftop photovoltaic (PV) installations, the observed series may reflect net-load effects. However, conventional consumers and prosumers are not separately identified in the available record, and no claim of separate prosumer modelling is made in this paper.

**Figure 1:**
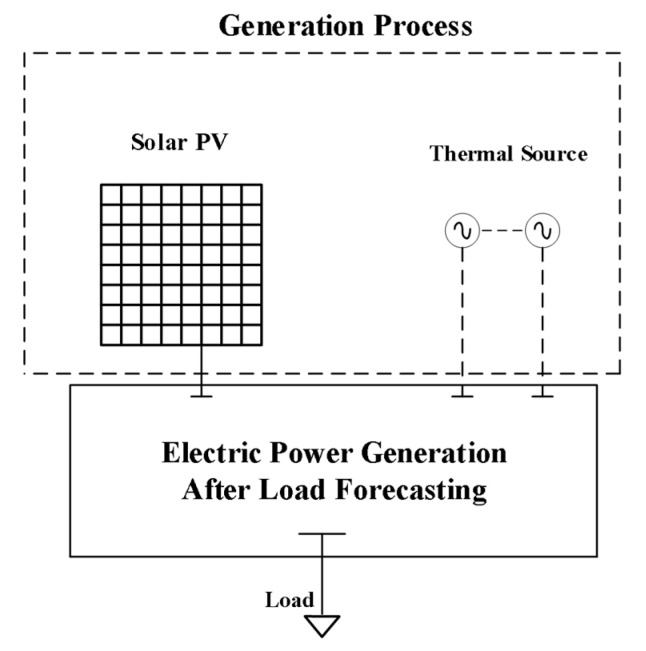
Overview of the power network containing consumers and prosumers.

Figure 1 presents an overview of the generalized power system of the central region of Pakistan, considering power produced through conventional thermal resources and the gradually increasing number of rooftop solar PV systems. The efficient operation of the LESCO network, which is going through a transitional phase due to the increasing number of prosumers, requires precise load forecasting.

This study makes four contributions:

- First, it provides an internally consistent daily-resolution evaluation of statistical, decomposition-based and deep hybrid frameworks for residential feeder load forecasting in the LESCO region, in which every framework is fitted to the same target series under the same chronological partition.
- Second, it gives a complete and reproducible formulation of the CWT-LeNet-5-LSTM architecture, in which multi-scale wavelet coefficients are processed by convolutional layers and reshaped into recurrent time steps, and it isolates the contribution of each component through an architecture ablation.
- Third, it compares all frameworks under identical exogenous calendar information and a single multi-lead forecasting protocol, against persistence and seasonal-naive references.
- Fourth, it reports errors stratified by season, by weekday and weekend, and by high, medium and low-load days. It also tests whether pairwise differences in accuracy are statistically significant.

### 1.2. Paper Outline

Section 2 presents a detailed literature review of load forecasting techniques, including the identification of the research gap, and a review of model-integrated and physics-informed learning, field-data generalisation and adaptation to drifting load distributions. Section 3 provides an overview of the application of ML and DL techniques for load estimation, gives a complete formulation of the proposed multi-scale hybridisation, and defines the precision testing techniques. Section 4 elaborates the data acquisition mechanism through the installed energy meters, the dataset specification, the feature construction and the evaluation protocol. Section 5 presents the results, including the architecture ablation, the stratified error analysis and the significance tests. Section 6 discusses the findings against the state of the art and interprets them in terms of the physical drivers of the LESCO load. Section 7 presents the conclusion.

## 2. Literature Review and Research Gap Identification

The authors in [20] executed an IoT-based smart metering mechanism for the real-time monitoring of electricity consumption of industrial users in Pakistan. The implemented framework was designed to facilitate power quality analysis, load profiling and automated billing, and demonstrated enhanced grid visibility through real-time analytics of power demand, demand-side management (DSM) and power factor correction. Reference [21] presented a personalised federated mechanism with privacy preservation for performing load estimation from data accumulated by smart meters. The designed mechanism managed noisy data so as to minimise latency in power demand estimation.

In [22], the authors presented an ML- and DL-based statistical evaluation of large datasets for accurate load estimation across various horizons and aggregation levels. The authors stated that although computational requirements varied significantly, the TFT and TiDE models consistently surpassed traditional mechanisms in forecasting accuracy.

Reference [23] elaborated on a smart domestic energy metering mechanism that combines non-invasive voltage and current instruments with an LSTM-based prediction model for continuous energy supervision and overload safety. A comparative analysis of GRU, LSTM, Random Forest and a hybridised CNN-LSTM mechanism is presented in [24] for short-term load forecasting; CNN-LSTM obtained the highest accuracy. CNN variants are usually employed in image processing and have shown improved performance in estimation when integrated with other techniques. Hybrid models that combine LSTM with CNN leverage the strengths of each method, resulting in more dependable predictions [25,26]. Table 1 shows a summary of the prominent neural networks employed for price and load estimation.

Load and price forecasting using hybridised methodologies such as ANN combined with LSTM networks has gained substantial attention because of its better performance in capturing both short-term needs and market dynamics [33,34]. Table 2 summarises the prominent ML/DL techniques hybridised with neural networks for load estimation.

Electricity cost and load demand prediction require innovative methodologies, such as the combination of GSA, FTS and LS-SVM [39]. Recent progress in prosumer-based electricity price and load estimation has seen substantial improvement with the rising use of data-driven methods to support the operation of distributed electric power mechanisms [40,41,42]. These methodologies assist in understanding complicated electricity cost and load patterns to provide precise estimations for prosumer-centric energy markets [43]. The incorporation of metaheuristic optimisation techniques further improves the performance of prediction mechanisms [44,45]. Similarly, the integration of fuzzy logic-based systems with ML and metaheuristic algorithms plays a significant role in precise forecasting while overcoming the non-linearities and uncertainties attributed to prosumer-based patterns [46,47].

### 2.1. Model-Integrated Learning, Field-Data Generalisation and Distribution Drift

The hybrid frameworks surveyed above combine established neural components, convolutional feature extractors, recurrent memories and signal decompositions. But they share a structural property that domain knowledge enters only through the choice of input representation, never through the learning objective itself. A parallel and more recent line of work embeds domain structure directly into the estimator. Physics-informed neural networks constrain the learned solution with the governing equations of the system, penalising the residual of a differential operator alongside the data misfit [48]. A closely related development in electrochemical storage is the Bayesian model-integrated neural network (BMINN) of Huang et al. [49], which infers electrode-specific chemical potentials and Gibbs free energies directly from current–voltage data without assuming a functional form for them, recovering staging structures and free-energy barriers that a purely data-driven regressor cannot resolve. The relevance of that work here is methodological rather than numerical. It shows that when a system possesses a known thermodynamic or physical structure, encoding that structure in the estimator yields both better extrapolation and interpretable latent quantities. Aggregated residential feeder demand has no comparably sharp governing equation, but it does possess exploitable structure, the astronomical annual cycle. Thermostatically driven cooling load and the deterministic calendar, and the exogenous Fourier and calendar block introduced in Section 4.4, encode that structure rather than requiring the network to rediscover it. The present study does not implement physics-informed learning, and no such claim is made.

A second limitation of the surveyed hybrids concerns generalisation from field data. Models validated on a single curated record frequently degrade when transferred to instrumentation, tariffs or usage regimes not represented in that record. Zhu et al. [50] addressed this directly for electric vehicle energy consumption by combining physics-informed features with an offline global model. A vehicle-specific online adaptation stage reduced the mean error of a quantile regression neural network from 6.30% to 5.04% and quantified the residual uncertainty rather than reporting a point estimate alone. The two-stage global-then-local structure was directly transferable to distribution feeders, where a single global model trained across many feeders could be adapted per feeder from a small number of local observations. The present study is confined to a single aggregated residential feeder and therefore cannot demonstrate such transfer; this is stated as a limitation in Section 6.5.

Third, the load distribution to which any of these models was fitted need not be stationary. Tariff restructuring, rooftop PV uptake and changes in occupancy are each a concept that drifts in the sense of Gama et al. [51]; the conditional distribution of demand given calendar covariates changes over time even when the marginal distribution does not covariate. A model selected once on a fixed historical window and never revisited will therefore decay silently. The recursive multi-step protocol adopted in Section 4.6 is a partial probe of this behaviour, since it forces each model to propagate its own errors across a lead without observing the truth. Full treatment requires online or continual adaptation, identified in Section 6.4 as the principal direction for future work. Battery-oriented studies, such as [48], are discussed here as methodological perspectives on incorporating domain structure into learning and are not proposed as direct forecasting benchmarks.

### 2.2. Research Gap

The largest portion (up to 50%) of electrical load in Pakistan is that of household consumers. Through the presented literature review, the following research needs were identified:

- The planning process depends on accurately forecasting electrical load for residential consumers connected to distribution feeders;
- There is a need for mechanisms that can analyse large utility datasets whose values may be noisy or incomplete because of the data collection practices of the distribution company;
- Existing comparisons frequently evaluate statistical and deep learning models under inconsistent forecasting protocols;
- One-step-ahead and multi-step recursive accuracy are not comparable quantities, and conflating them can invert the apparent ranking of competing models;
- Existing comparisons frequently supply unequal information to the competing models. Withholding calendar regressors from a SARIMAX baseline while a deep model infers calendar structure implicitly from a long input window overstates the benefit of the deep model;
- Evidence remains limited on whether multi-scale wavelet–convolutional–recurrent models improve residential feeder forecasts across multiple leads. When strong baselines receive the same information, which component of such a hybrid model produces the observed improvement?

## 3. Overview of Statistical, DL and Hybrid Algorithms and Mathematical Modelling

**Figure 2:**
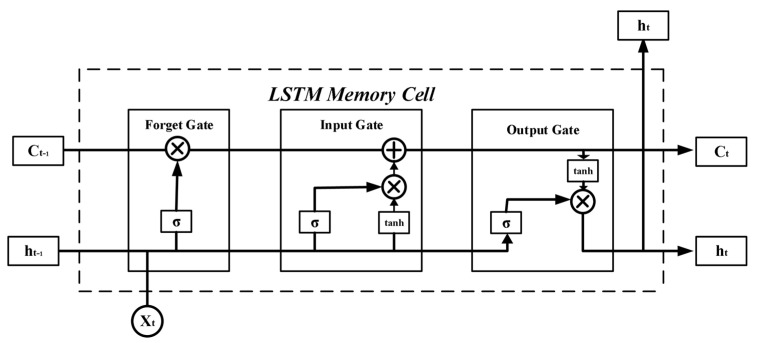
Generalised representation of the cell structure of the LSTM [52].

Deep learning techniques such as LSTM, and statistical techniques such as SARIMA and its exogenous extension SARIMAX, are used to capture dependencies in sequential data, which makes them suitable for time-series tasks such as load estimation. Figure 2 presents the cell structure of the LSTM, which is used in this study in both its original and hybridised forms.

The gate-level recurrence of a standard LSTM cell, the forget, input and output gates, and the cell state update are standard material and are not reproduced as in [52].

**Figure 3:**
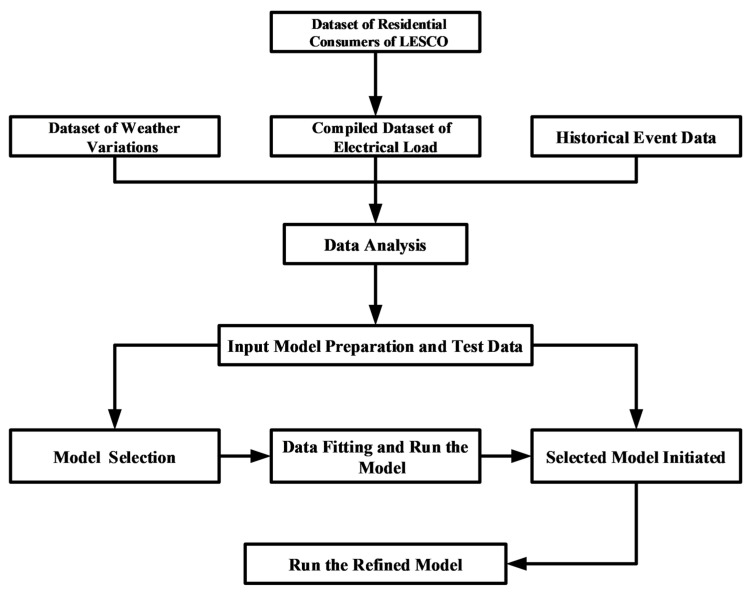
Time-series analysis-based load forecasting model.

Figure 3 presents the general time-series analysis-based load forecasting model, in which historical demand together with calendar and event information is filtered, model parameters are selected and the forecasting algorithm is executed.

An important aspect of this study is that only the historical electrical load and the deterministic calendar are available in the analysed record. No weather or event measurements accompany the data, as recorded in Table 3, and none are used by any model in this study.

### 3.1. Forecasting Problem Formulation

The forecasting mechanism is formulated as a non-linear autoregressive problem with exogenous inputs. Let *y_t* denote the residential feeder load on day t, let *L* = 128 be the length of the input window and let *h* be the forecast lead. The task is,

(1) $${\hat{y}}_{t+h}=f_{\theta }\left(y_{t-L+1:t},z_{t+h}\right)+\varepsilon_{t+h}$$

Here, z is the exogenous calendar vector for the target day,

(2) $$z_{t}={\left[c_{1},\;c_{2},\;c_{3},\;c_{4},\;sin\frac{2\pi w_{t}}{7},\;cos\frac{2\pi w_{t}}{7},\;sin\frac{2\pi m_{t}}{12},\;cos\frac{2\pi m_{t}}{12},\;1\left[w_{t}\geq 5\right],\;\tau_{t}\right]}^{⊤}\;c_{k}=\left[sin\frac{2\pi kd_{t}}{365.25},\;cos\frac{2\pi kd_{t}}{365.25}\right],\;k=1,\ldots ,4$$

in which *d_t* is the day of year, *w_t* the day of week (Monday = 0), *m_t* the month and *tau_t* a linear time index in years since the start of the record. Four annual harmonics were retained; adding a fifth did not reduce validation error. The indicator term marks Saturdays and Sundays. The vector therefore has 14 components: eight annual Fourier terms, two day-of-week terms, two-month terms, one weekend indicator and one trend term. This same vector is supplied to every model considered in this study, so that the statistical and deep learning frameworks receive identical calendar information.

The SARIMAX specification used as the statistical baseline is,

(3) $$\phi_{p}\left(B\right){\left(1-B\right)}^{d}\left(y_{t}-\beta^{⊤}z_{t}\right)=\theta_{q}\left(B\right)\varepsilon_{t},\;\varepsilon_{t}∼WN\left(0,\sigma^{2}\right)$$

where *B* is the backshift operator, *φ* and *θ* are the autoregressive and moving-average polynomials of orders *p* and *q*, and *d* is the order of differencing. The annual cycle is carried by the Fourier terms in *z* rather than by a seasonal ARMA polynomial. A seasonal period of m = 365 is computationally intractable in a state-space representation.

### 3.2. Precision Analysis

Mean absolute error (MAE), root mean square error (RMSE) and mean absolute percentage error (MAPE) are used to authenticate the accuracy of the forecasted values [51,52]. For n test points with actual values *A* and estimated values *E*,

(4) $$MAE=\frac{1}{n}\sum_{i=1}^{n}\left|A_{i}-E_{i}\right|$$

(5) $$RMSE=\sqrt{\frac{1}{n}\sum_{i=1}^{n}{\left(A_{i}-E_{i}\right)}^{2}}$$

(6) $$MAPE=\frac{100}{n}\sum_{i=1}^{n}\left|\frac{A_{i}-E_{i}}{A_{i}}\right|$$

MAE and RMSE are expressed in kilowatts, the unit of the target series; only MAPE is a percentage; and the “loss percentages” is the absolute errors divided by the peak training load. The normalisation is retained here but is now named and defined explicitly as the peak-normalized RMSE, so that the reported percentages can be reproduced from the absolute figures,

(7) $$nRMSE=\frac{100}{y_{max}^{train}}\sqrt{\frac{1}{n}\sum_{i=1}^{n}{\left(A_{i}-E_{i}\right)}^{2}}$$

where the denominator is the maximum load observed over the training span, 9998 kW. Because two models can differ in mean error without that difference being statistically meaningful on a finite test set, pairwise accuracy differences are tested with the Diebold–Mariano statistic [53],

(8) $$DM=\frac{\bar{d}}{\sqrt{\hat{Var}\left(\bar{d}\right)}},\;d_{i}={\left(A_{i}-E_{i}^{\left(1\right)}\right)}^{2}-{\left(A_{i}-E_{i}^{\left(2\right)}\right)}^{2}$$

It is computed on squared-error loss differentials at a fixed lead, from identical forecast origins and identical targets, with a heteroskedasticity and autocorrelation-consistent variance estimate truncated at that lead.

### 3.3. Formulation of the Proposed Multi-Scale Hybridisation

This subsection gives the explicit tensor algebra of the proposed CWT-LeNet-5-LSTM model and explains how the wavelet coefficients of the load window are arranged, how they pass through the two-dimensional convolutional filters of the LeNet-5 stage, and how the resulting feature maps are flattened into the recurrent time steps of the LSTM.

#### 3.3.1. Continuous Wavelet Transform Stage

The continuous wavelet transform of the input window *x* is,

(9) $$W_{x}\left(a,b\right)=\frac{1}{\sqrt{a}}\int_{-\infty }^{\infty }x\left(u\right)\;\psi^{*}\left(\frac{u-b}{a}\right)du$$

with *a* representing the scale, *b* the translation and *Ψ* the mother wavelet. The implementation uses the Morlet wavelet of the PyWavelets (version 1.7.0) library, invoked as the “morl” continuous wavelet, whose real-valued kernel is,

(10) $$\psi \left(u\right)=\pi^{-1/4}e^{i\omega_{0}u}e^{-u^{2}/2},\;\omega_{0}=5$$

The Morlet kernel was selected over the Mexican-hat and Daubechies families because its scale-to-period mapping is interpretable in days, which makes the resulting scalogram physically readable [54].

Thirty-two integer scales are evaluated, *a* = 1, 2,..., 32, which covers the sub-weekly, weekly and monthly bands while remaining inside the cone of influence of a 128-day window. The magnitude of the transform is retained, giving the real-valued scalogram,

(11) $$S_{s,l}^{\left(t\right)}=\left|W_{x_{t}}\left(a_{s},l\right)\right|,\;S^{\left(t\right)}\in R^{32\times 128},\;a_{s}=s,\;\;s=1,\ldots ,32$$

So, each 128-day load window is represented as a single-channel image of 32 rows (scales) by 128 columns (time). Scalograms are standardized with only the mean and standard deviation of the training split.

#### 3.3.2. LeNet-5 Convolutional Stage

The scalogram is processed by two LeNet-5 [55] convolution-pooling blocks. The first applies six 5 × 5 kernels with unit stride and the “same” zero padding, followed by a hyperbolic tangent non-linearity,

(12) $$H_{c}^{\left(1\right)}=tanh\left(\sum_{c^{\prime }}K_{c,c^{\prime }}^{\left(1\right)}*S_{c^{\prime }}+b_{c}^{\left(1\right)}\right),\;c=1,\ldots ,6$$

where the convolution runs jointly over the scale and time axes so that each kernel responds to a localized time–frequency motif rather than to an instantaneous amplitude. Pooling is anisotropic, with the average pooling having a 2 × 1 window and stride (2,1) halving the scale axis while leaving the time axis untouched,

(13) $$P_{c}^{\left(1\right)}\left[s,l\right]=\frac{1}{2}\sum_{u=0}^{1}H_{c}^{\left(1\right)}\left[2s+u,\;l\right]$$

Conventional LeNet-5 pools symmetrically in both spatial dimensions, which for a scalogram would reduce exactly the temporal resolution that the recurrent stage consumes.

Preserving the time axis at full length means the convolutional stack acts as a learned, scale-mixing filter bank applied pointwise in time, and the sequence handed to the LSTM retains one element per day. This design choice is examined empirically in the ablation of Section 5.7. The second block repeats the operation with sixteen 5 × 5 kernels, giving the overall progression.

(14) $$S\in R^{1\times 32\times 128}\;\to \;P^{\left(1\right)}\in R^{6\times 16\times 128}\;\to \;P^{\left(2\right)}\in R^{16\times 8\times 128}$$

#### 3.3.3. Flattening into Recurrent Time Steps

The rank-three tensor produced by the convolutional stack is permuted so that the time axis leads, and the remaining channel and scale axes are collapsed into a single-feature axis,

(15) $$u_{l}=vec\left(P^{\left(2\right)}\left[:,:,l\right]\right)\in R^{128},\;l=1,\ldots ,128$$

yielding a sequence of 128 vectors, one per day of the input window, each of dimension 16 channels × 8 residual scales = 128. No information is aggregated across time at this point; the flattening is purely a re-indexing.

#### 3.3.4. Recurrent Stage and Exogenous Fusion

The sequence is consumed by a single LSTM layer with 64 hidden units,

(16) $$\left(h_{l},c_{l}\right)=LSTM_{\vartheta }\left(u_{l},h_{l-1},c_{l-1}\right),\;h_{l}\in R^{64}$$

And the terminal hidden state is concatenated with the calendar vector of the target day before the dense head,

(17) $${\hat{y}}_{t+1}=w_{2}^{⊤}\;ReLU\left(W_{1}\left[h_{128};\;z_{t+1}\right]+b_{1}\right)+b_{2}$$

**Figure 4:**
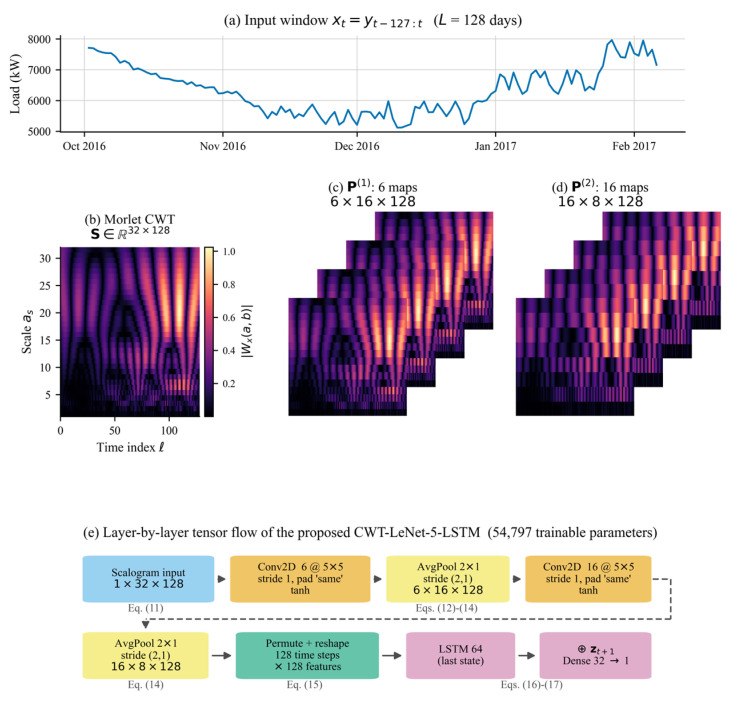
Architecture of the proposed CWT-LeNet-5-LSTM model. (**a**) A representative 128-day input window; (**b**) its Morlet scalogram; (**c**,**d**) feature maps after the first and second convolution-pooling blocks; (**e**) layer-by-layer tensor flow with kernel sizes, strides and output shapes, cross-referenced to Equations (9)–(17).

Late fusion of the calendar block is used rather than early concatenation to the scalogram, because the calendar variables are deterministic functions of the target date and carry no time–frequency content for the wavelet stage to decompose. Dropout [56] with rate 0.2 is applied to the terminal hidden state. The complete model has 54,797 trainable parameters. Figure 4 illustrates the corresponding tensor shapes at each stage, and the different colours of blocks (at the bottom of the figure) are used to distinguish the equations used in the respective operation of the framework.

## 4. Research Methodology

The process begins with consumer data collection, keeping in view geographical location and way of living because these affect the consumption of electricity. The dataset is segregated according to the category of load, such as general residential consumers and modern-era users who partially generate their own electricity through rooftop solar PV systems.

### 4.1. Data Acquisition Through the LESCO Distribution System

#### 4.1.1. Data Collection Through Three-Phase Energy Meters

**Figure 5:**
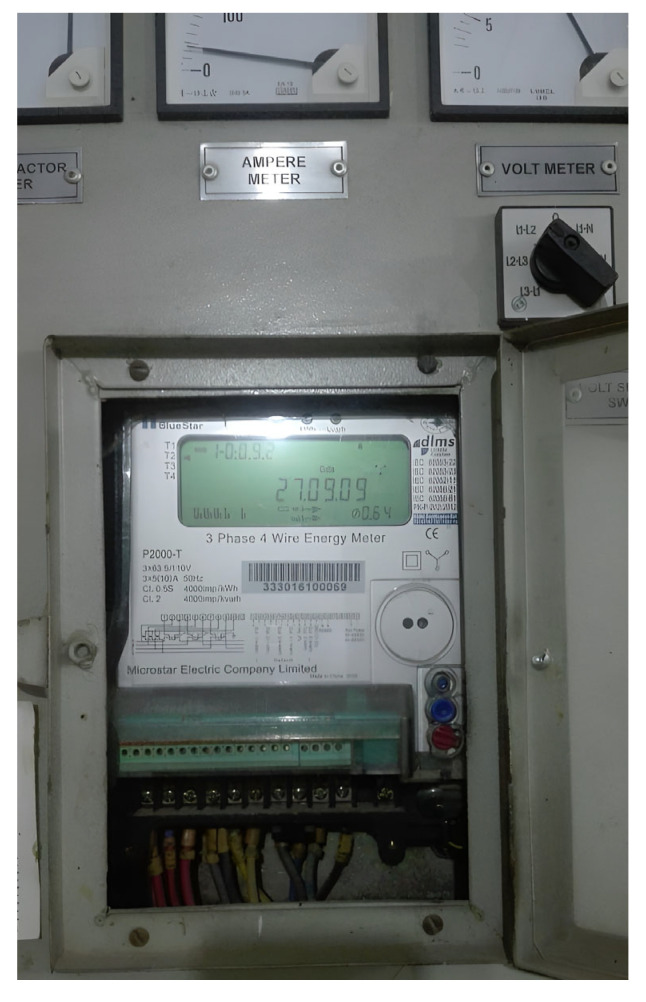
Close-up view of the sensor-based feeder metering infrastructure used for data acquisition in the proposed load forecasting framework.

Figure 5 presents the three-phase four-wire energy meter, based on various sensors and electronic circuitry, installed at the grid station on the outgoing 11 kV distribution feeder of LESCO, Pakistan. These meters act as the primary source of the data used in this study and are built around instrument-grade CTs, direct voltage sensing circuits or PTs, and high-resolution ADCs. The meters also embed DSP algorithms operating on the information received from the integrated sensors, an RTC, a microprocessor-based metering unit, an optical communication interface supporting DLMS/COSEM (IEC 62056) [57] protocols, and non-volatile memory.

These sensors continuously collect synchronised three-phase currents and voltages for the computation of kWh, kVARh, kVAh, kW, kVAR and kVA. Fundamental parameters such as phase currents and voltages, frequency, maximum demand and power factors are also recorded with utility-grade accuracy in accordance with IEC 62053 [58] and IEC 62052-11 [59].

#### 4.1.2. Data Collection Through Single-Phase Energy Meters

**Figure 6:**
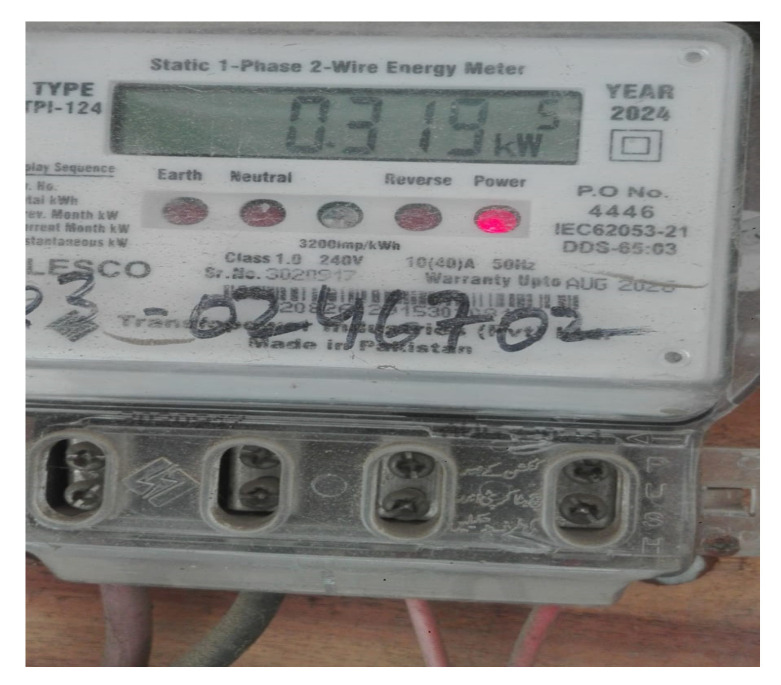
Domestic single-phase electronic energy meter deployed by LESCO for data acquisition.

Distributed generation modifies domestic power characteristics by reducing daytime net demand, producing reverse power flow during periods of excess production, and increasing demand variability. Figure 6 shows an electronic sensor-based single-phase energy meter installed at the premises of domestic consumers within the service territory of LESCO.

This is a class 1.0 energy meter with embedded electronic sensors, compliant with IEC 62053-21 [59], consisting of current and voltage sensing circuits, an analogue front end, energy metering ICs and ADCs, together with non-volatile memory and an embedded microprocessor for digital signal processing.

### 4.2. Dataset Specification

The specifications below are used by every model reported in this paper, and Table 3 states it in full.

The recorded quantity is in kW, the target column name is “Residential_Plot” and no monthly aggregation is performed at any point.

### 4.3. Load Characteristics and Choice of Seasonal Period

**Figure 7:**
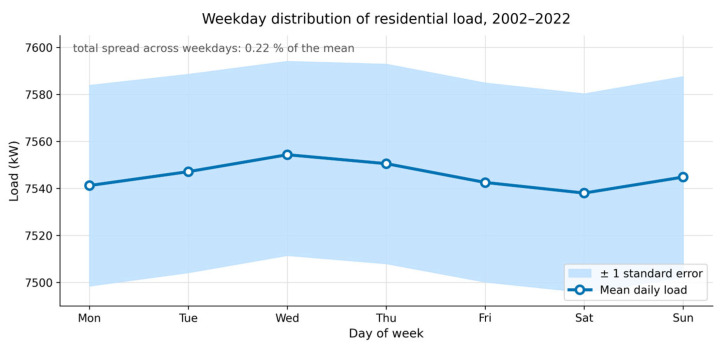
Weekday distribution of load for residential consumers using the collated dataset.

Figure 7 shows the weekday distribution; the spread between the highest and lowest weekday means is only 0.22% of the series mean. After first differencing, the autocorrelation at lag 7 is 0.012, indistinguishable from the neighbouring lags and well inside the 95% white-noise band, so no weekly component survives the removal of the trend. The dominant periodicity is annual, and the sample autocorrelation at lag 365 is 0.80. The monthly means range from 5669 kW in December to 9273 kW in July, a spread of 47.8% of the mean and all seasonal specifications are therefore annual.

**Figure 8:**
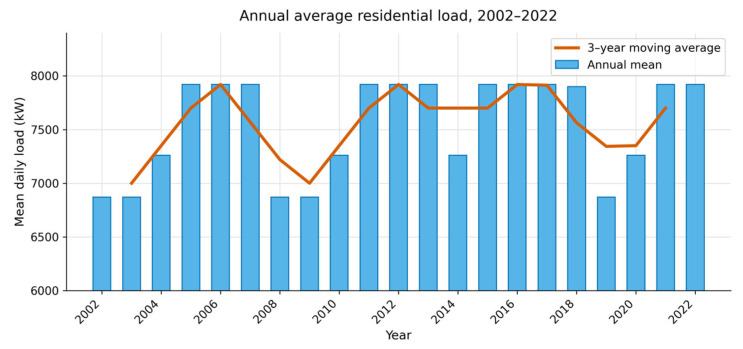
Annual average distribution of load among residential consumers using the collated dataset.

As presented in Figure 8, the annual average pattern shows the load fluctuating within a band of roughly 6900 to 7900 kW across the record, without a monotonic growth trend. An important point to notice is that, despite a steady increase in the number of new households, the mean residential demand on this feeder does not increase correspondingly. A plausible contributing factor is the local generation of electricity by domestic consumers who have installed rooftop solar systems in response to rising electricity prices and frequent outages. The available record does not contain PV capacity or export measurements, so this explanation is offered as context and is not quantified by the present analysis.

**Figure 9:**
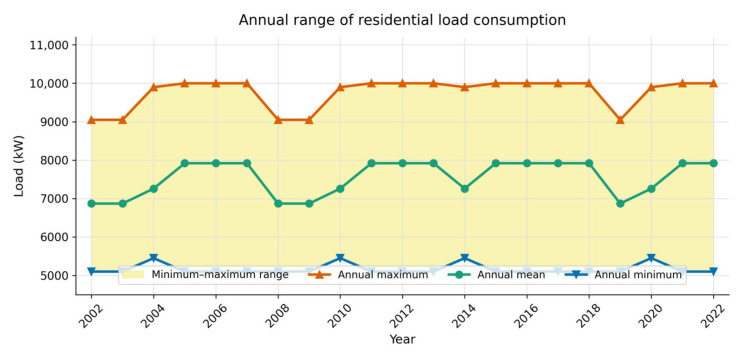
Annual range of load consumption using the collated dataset.

As presented in Figure 9, the maximum demand remains just below 10,000 kW, while the annual mean ranges between roughly 6900 and 7900 kW, and the annual minimum stays close to 5000 kW. The duration of peak demand is shorter for the minimum series than for the average series, reflecting the sharper summer excursions.

**Figure 10:**
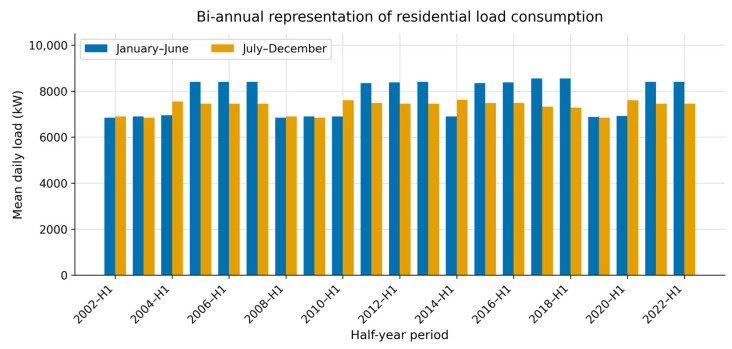
Bi-annual data representation of load consumption for the considered period using the collated dataset.

During the summer half-year, the load is markedly higher because of cooling demand, while in the winter half-year, the demand is lower, which highlights the seasonality trend that dominates this series, as presented in Figure 10.

**Figure 11:**
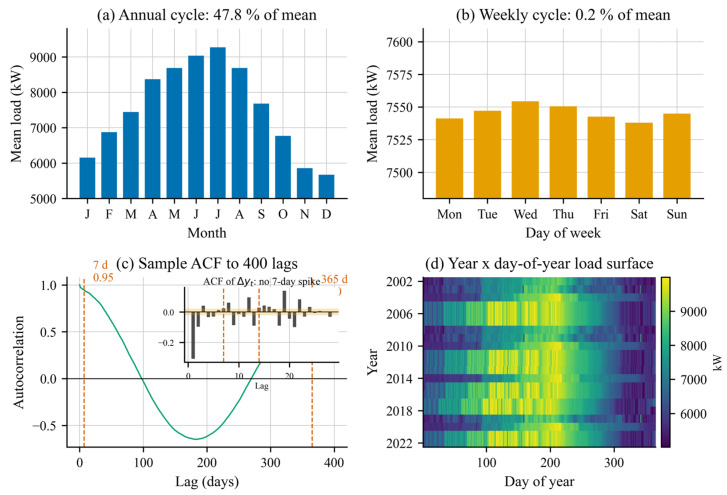
Seasonality evidence. (**a**) Mean load by calendar month; (**b**) mean load by day of week on an expanded vertical scale; (**c**) sample autocorrelation to 400 lags, with the inset showing that no seven-day spike survives first differencing; (**d**) year × day-of-year load surface.

Figure 11 presents the seasonal evidence for the monthly load (depicted using the initial letter of the month) along-with the daily loads as well. Additionally, the figure also presents the variation graph for autocorrelation vs lag (days) to highlight the variation of the inset. The surface chart for the yearly load variation according to the 365 days of an year is also presented for the period of 2002–2022.

### 4.4. Feature Construction

In the presented study, the calendar block defined in Equation (2), comprising 14 variables, is supplied to every model. For SARIMAX, it enters as the exogenous regressor matrix. For the recurrent models, it is concatenated channel-wise to the load channel at every step of the input window, giving one endogenous + 14 exogenous channels, for a total of 15 input channels. The proposed hybrid model is fused late at the dense head, as described in Section 3.3.4. The gradient boosting residual learner additionally receives lagged loads at 1, 2, 3, 7, 14, 28 and 365 days and rolling means and standard deviations over 7, 28 and 90 day windows, all constructed from strictly past observations.

Making the calendar block available to SARIMAX is what allows the statistical baseline to be a fair comparator. Table 4 reports the fitted exogenous coefficients with the first annual harmonic dominating, while the day-of-week and weekend terms are individually insignificant, which corroborates the seasonality analysis of Section 4.3 from a second direction.

### 4.5. Training, Validation and Test Protocol

**Figure 12:**
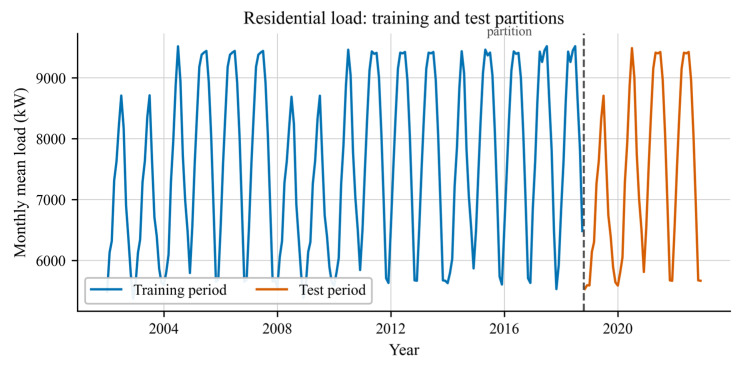
Annual range of electrical load consumption, training versus test partitions, using the collated dataset.

The series is partitioned chronologically. The first 5216 days (1 January 2002 to 12 April 2016) form the fitting set; the following 920 days (13 April 2016 to 19 October 2018) form the validation set. The final 1534 days (20 October 2018 to 31 December 2022) form the test set, which is held out entirely until the final evaluation. No shuffling is performed at any stage, and the test set is never used for architecture selection, hyperparameter tuning, early stopping or feature selection. All scaling parameters are estimated on the fitting split alone and applied unchanged to the validation and test splits, while the forecasts are inverted to kilowatts before any metric is computed. Figure 12 presents the annual range of electrical load consumption and training versus test partitions, using the collated dataset.

The original LSTM is trained for a fixed 1000 epochs on 140 sequences with neither a validation set nor early stopping, which is an overfitting risk, and it is addressed as follows:

- First, modelling the series at daily rather than monthly resolutions increases the number of training sequences from 140 to 5088.
- Second, training is governed by early stopping on validation loss with a patience of 15 epochs and restoration of the best weights. Together with a learning-rate reduction by a factor of 0.5 after six stagnant epochs, the epoch count is an upper bound rather than a target. The four-layer LSTM reached its best validation loss at epoch 30 and was stopped at epoch 45.
- Third, a capacity ablation over depth and width is reported in Section 5.1. Dropout of 0.2 is retained on all recurrent and dense stages, and gradients are clipped to unit norm. Because a single run of a stochastic optimizer is not a reliable point estimate, every deep framework was retrained under three random initialisations and is reported as the mean and standard deviation across those runs.
- The dashed line differentiating the ending of training period and starting of the testing period.

### 4.6. Evaluation Protocol

In this study, every framework is evaluated under one recursive protocol. From non-overlapping origins spaced h days apart, a model produces an *h*-day trajectory by feeding its own predictions back as inputs. Without observing any actual load inside the trajectory, only the deterministic calendar regressors, which are known in advance, are supplied. Errors are then pooled over all leads within the trajectory, and to avoid ambiguity, the results are labelled by lead. Lead-1, lead-7, lead-14 and lead-30 accuracy refer to trajectories of 1, 7, 14 and 30 days, respectively, each pooled over the whole trajectory. Lead 1 reduces to one-step-ahead forecasting with a fully observed history, corresponding to day-ahead scheduling. Lead 30 corresponds to monthly operational planning, which is the setting of the intended multi-scale hybrid. All frameworks share the same forecast origins and the same targets at every lead.

Two naive references are reported alongside the five frameworks: persistence, which carries the last observed value forward across the trajectory, and a seasonal naive forecast, which predicts the value observed 365 days earlier. A forecasting model that does not beat both of these under the protocol in which it is deployed has not demonstrated value.

**Figure 13:**
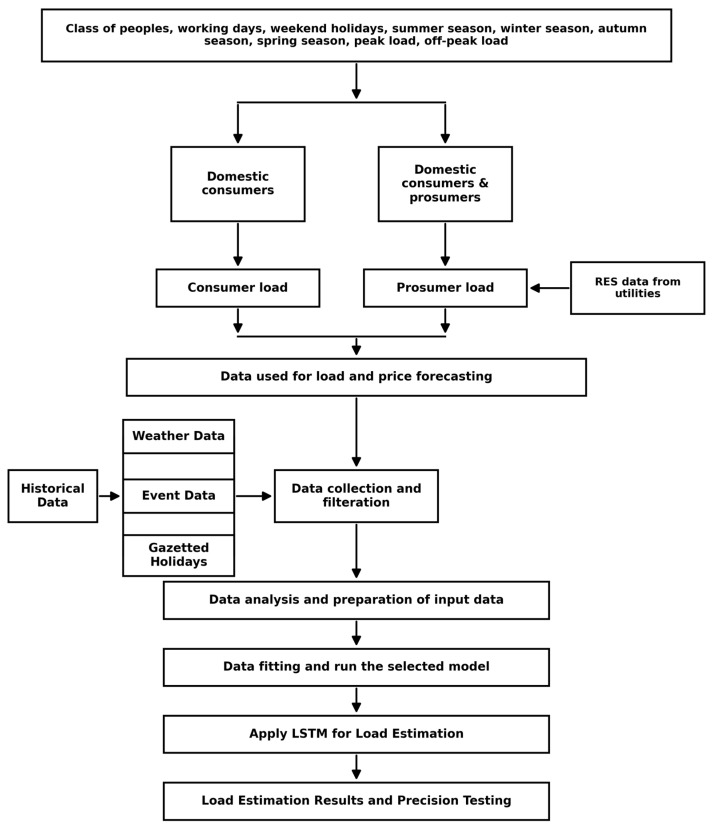
Flowchart of the designed research approach.

Figure 13 presents the flowchart and describes the intended general procedure. Two of its branches could not be realized through the available record:

- The weather, event and gazetted-holiday inputs are not present in the data and are not used.
- Consumer and prosumer loads are not separately metered in the record.

The target is the aggregate residential feeder series, which may embed net-load effects from customers with rooftop PV systems but does not permit the two categories to be separated. The path actually executed in this study runs from the historical feeder load and the calendar block, through the partition of Section 4.5, to the five frameworks and the precision testing of Section 3.2.

### 4.7. Reproducibility Settings

In particular, the mother wavelet and scale count of the CWT and the kernel sizes and strides of the LeNet-5 stage were investigated. Table 5 lists every setting required to reproduce the study and presents the complete configurations of the five frameworks considered.

## 5. Results

All results below are produced by a single pipeline on the dataset and partition specified in Section 4.2, and every framework receives the exogenous block of Equation (2).

### 5.1. Training Behaviour and Model Capacity

**Figure 14:**
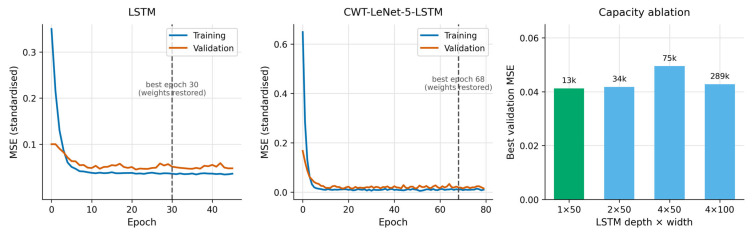
Training dynamics and capacity ablation. Training and validation loss for (**left**) the four-layer LSTM and (**centre**) the proposed CWT-LeNet-5-LSTM, with the restored epoch marked; (**right**) best validation loss against recurrent depth and width, annotated with parameter counts.

Figure 14 shows the training and validation curves for the plain LSTM and the proposed hybrid model, together with the capacity ablation. Two observations follow from Figure 14,

- First, the validation loss of the four-layer LSTM reaches its minimum at epoch 30 and rises thereafter, while the training loss continues to fall, which indicates overfitting. Early stopping restores the weights from the validation optimum, so the reported figures are those of the best-generalizing iterate.
- Second, validation error is essentially flat across depths of one to four layers at 50 units, and widening to 100 units does not help, so the capacity of the recurrent stack is not the binding constraint on this series.

### 5.2. SARIMAX

The SARIMAX order was selected on the validation span. The retained specification is (3, 1, 1) with the exogenous block and no seasonal ARMA polynomial, reaching a validation MAPE of 2.57%. The (1, 1, 1), (1, 1, 1, 7) specification of the original submission, without exogenous regressors, reaches 2.70% on the same validation span and a higher AIC, confirming that the weekly seasonal polynomial was not merely unsupported but worse than the annual Fourier parameterisation.

**Figure 15:**
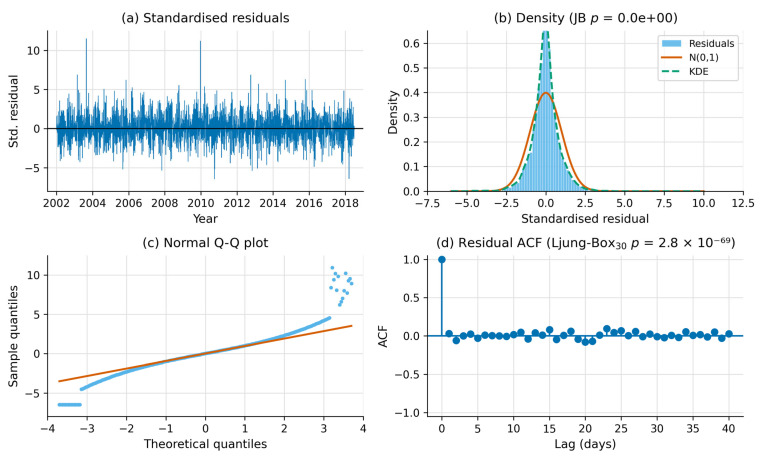
SARIMAX residual diagnostics. (**a**) Standardised residuals; (**b**) residual density against the standard normal with a kernel density estimate; (**c**) normal quantile-quantile plot; (**d**) residual autocorrelation with Ljung–Box *p*-value.

Figure 15 presents the standardized residuals against time, their density against the standard normal, a normal quantile–quantile plot, and the residual autocorrelation function. The Jarque–Bera statistic is 32,222 with excess kurtosis of 11.2, and the Ljung–Box statistic at lag 30 gives *p* < 0.001, so the residuals are neither normal nor serially independent. The seasonal banding visible in panel (a) shows that residual variance is larger in the summer months. These diagnostics are the formal justification for moving beyond a linear model where the structure remains in the residuals that a linear Gaussian specification cannot represent. The orange line in the panels (b) and (c) represent the reference theoretical standard, normal distribution *N* (0, 1) used for the comparison with the residuals. The blue dots in panel (c) show the individual standardised residual sample points. In panel (d) the blue dots represent the individual ACF values for specific lags.

**Figure 16:**
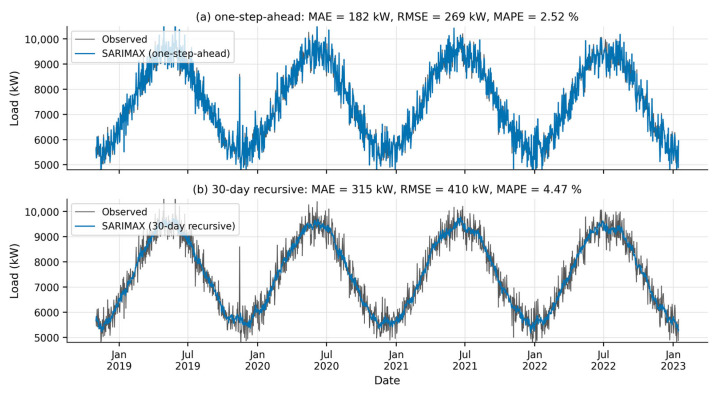
SARIMAX forecasting performance on the test span: (**a**) lead 1; (**b**) lead 30.

Figure 16 presents the actual forecasting performance of the fitted SARIMAX and is shown here at both extremes of the lead range.

The results indicate that SARIMAX models linear structure efficiently but struggles with abrupt shifts in demand and the amplitude of the summer peak, where its residual variance is concentrated.

### 5.3. SARIMA with Gradient Boosting

**Figure 17:**
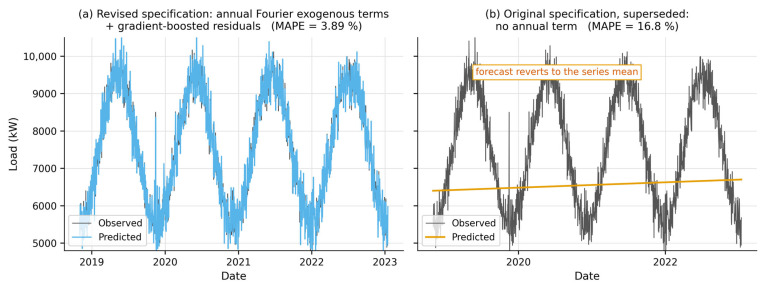
SARIMA with gradient boosting residual learning. (**a**) The revised specification, with annual Fourier exogenous regressors in the base model. (**b**) The original specification reproduced for comparison: a seven-day seasonal polynomial with no annual term, whose out-of-sample forecast reverts to the unconditional meaning of the series.

Once the annual cycle is carried by Fourier regressors in the base model, the same hybrid attains 3.89% at lead 30 and 0.76% at lead 1. Figure 17 shows the performance of the SARIMA-GB model for the considered period in panel (a) and the superseded original in panel (b).

The gradient boosting stage has access to the load of the preceding day, and on a series whose lag-one autocorrelation is 0.978, that single feature carries most of the achievable one-step accuracy. The resulting MAPE of 0.76% is the lowest of any framework in this study, but it indicates that one-step-ahead prediction on a smooth daily series is an easy problem rather than the hybrid framework that has learned the dynamics of the feeder. The longer leads in Table 6, where the same model is denied that feature, are more informative comparatively.

### 5.4. STL Decomposition with LSTM

**Figure 18:**
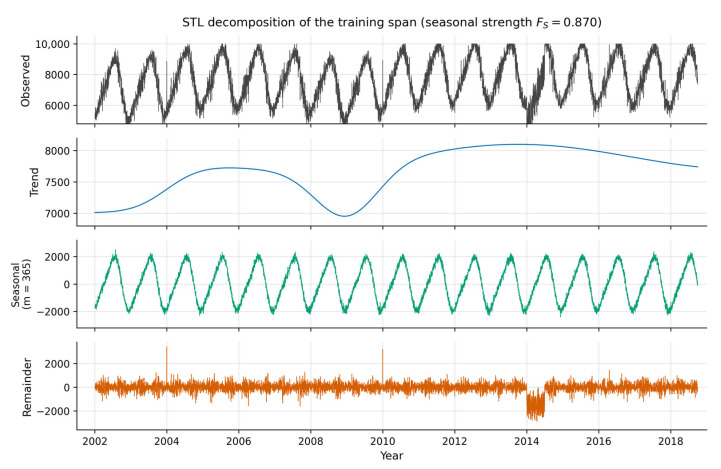
STL decomposition of the training span with an annual period, showing the observed series, the extracted trend, the seasonal component and the remainder.

STL decomposition with LSTM is used as the second integrated mechanism, separating the load series into trend, seasonal and remainder components and modelling the remainder with a recurrent network. The decomposition period is 365 days rather than the seven days used in the original submission, for the reasons established in Section 4.3, and the decomposition is fitted only to the training span, so that no future information enters the components. Figure 18 presents the STL decomposition of the training span with an annual period, showing the observed series, the extracted trend, the seasonal component and the remainder.

The dark gray line represents the original, raw load time-series data while the blue line represents the long-term underlying direction and low-frequency movement of the series after seasonal and irregular variations are removed. The green line represents the recurring intra-year seasonal pattern with an annual period of 365 days, and the orange line represents the residual noise and irregular fluctuations (including random shocks and sporadic spikes) left over after removing the trend and seasonal components.

The decomposition confirms that the seasonal component accounts for the greater part of the variance and that the remainder retains substantial structure. Constraining the LSTM to the remainder signal removes its access to cross-scale interactions, which is the likely reason this hybrid does not improve on the plain multivariate LSTM at any lead.

### 5.5. LSTM

The load channel is accompanied at every step of the 128-day input window by the 14 exogenous calendar channels of Equation (2). This is a direct response to the observation that excluding calendar features undermines the baselines, and the effect is substantial. The standard LSTM becomes the strongest non-wavelet deep framework, reaching 3.32 ± 0.37% at lead 30.

### 5.6. CWT-LeNet-5-LSTM

**Figure 19:**
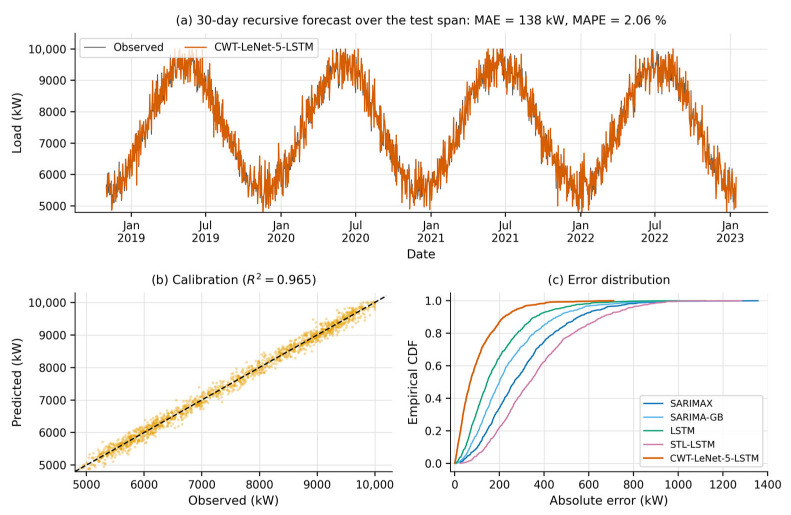
Forecasting performance of the CWT-LeNet-5-LSTM model. (**a**) Lead-30 recursive forecast across the test span; (**b**) predicted against observed load; (**c**) empirical distribution of absolute errors for all five frameworks.

Figure 19 shows the Forecasting performance of the CWT-LeNet-5-LSTM model, with the results for Lead-30 recursive forecasts across the test span, predicted against the observed load, and the empirical distribution of absolute errors presented. CWT provides a multi-resolution time-frequency decomposition of the load signal, the LeNet-5 stage extracts salient time-frequency features, and the LSTM layer captures long-range temporal dependencies from the resulting representations. The black dashed line represents the ideal 1:1 reference line (y=x) where predictions perfectly match observations, while the orange scatter points display the actual model predictions plotted against true values. The parameters of each stage are specified in Section 3.3 and Table 5, and the tensor flow is shown in Figure 4.

### 5.7. Architecture Ablation

The improvement claimed for the proposed framework is attributed to four design elements:

- The wavelet representation;
- The convolutional stage;
- The anisotropic pooling that preserves temporal resolution;
- The late fusion of the calendar block.

To establish which of these actually contributes, Table 6 reports variants in which one element at a time is removed or replaced. All variants were trained under the same partition, optimizer and early stopping rule with a single common seed. Table 6 shows the architecture ablation, with each variant differing from the proposed model by one element. Lead-1 and lead-30 accuracy on the test span, single seed.

**Table 6:** Analysis of lead 1 and 30 achieved through variants of CWT-LSTM and CWT-LeNet-5-LSTM.

Variant	What It Isolates	Params	Lead-1 MAPE (%)	Lead-30 MAPE (%)
CWT-LSTM (no convolution)	Value of the convolutional stage	27,649	1.39	3.13
CNN-LSTM (no wavelet)	Value of the wavelet representation	24,085	2.48	5.00
CWT-LeNet-5-LSTM, symmetric pooling	Value of anisotropic pooling	54,797	2.25	3.53
CWT-LeNet-5-LSTM, no calendar fusion	Value of the calendar block	54,349	1.28	3.10
CWT-LeNet-5-LSTM (proposed)	Full model	54,797	1.15	2.31

Removing the wavelet stage and convolving the raw window raises the lead-30 MAPE from 2.31% to 5.00%, and removing the convolutional stage while retaining the scalogram raises it to 3.13%. So, the two stages contribute jointly rather than either being redundant. Replacing the anisotropic 2 × 1 pooling with conventional symmetric 2 × 2 pooling raises the lead-30 MAPE to 3.53% and the lead-1 MAPE from 1.15% to 2.25%, which is the empirical basis for preserving the time axis through the convolutional stack. Removing the calendar fusion raises lead-30 MAPE to 3.10%, confirming that the exogenous block contributes to the multi-step result and is not merely redundant with the information already present in the input window.

### 5.8. Comparative Analysis

Table 7 reports every framework at lead 1 and lead 30; Table 8 reports all four leads, and SARIMAX reports an MAE of 21.44% against an RMSE of 16.67%. Table 7 presents the test set accuracy for all frameworks, where columns 2–5 are for lead 1 and columns 6–9 are for lead 30. MAE and RMSE are in kilowatts, and nRMSE is normalized by the training peak of 9998 kW. Deep frameworks are shown for the reference seed, with the variability across seeds given in Table 7, and the shaded row is the best framework at lead 30.

The dependence on the forecast lead is the central comparative finding of this study, and it is why Table 8 is reported rather than a single number. At lead 1, the ranking is led by SARIMA-GB (0.76%), because the previous day’s observed load is available and dominates the prediction.

**Figure 20:**
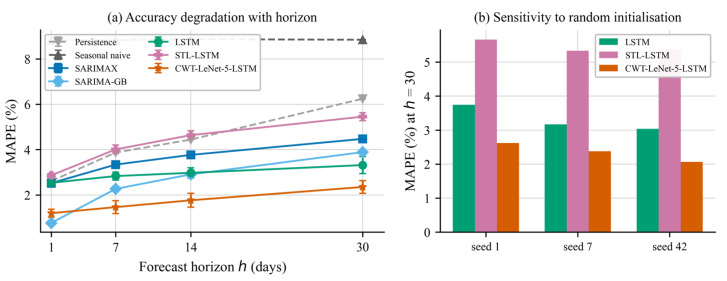
(**a**) MAPE against forecast lead for all frameworks, with error bars showing the standard deviation across 3 random initialisations for the deep frameworks, (**b**) sensitivity of the deep frameworks to random initialisation at lead 30.

Persistence itself achieves 2.61%, so this lead has limited power to discriminate between frameworks. As the lead lengthens, the spread widens and the ordering changes by lead 30; CWT-LeNet-5-LSTM leads with 2.35 ± 0.28% against 3.32% for the multivariate LSTM. SARIMA-GB achieves 3.89% and 4.47% for SARIMAX, while persistence degrades to 6.24%, with the degradation curves presented in Figure 20a.

Figure 20b presents the outcomes for each deep framework under three random initialisations and produces a spread that is not negligible relative to the differences between frameworks. A single run would therefore overstate the precision of the comparison, and the standard deviations in Table 8 should be read alongside the means.

**Figure 21:**
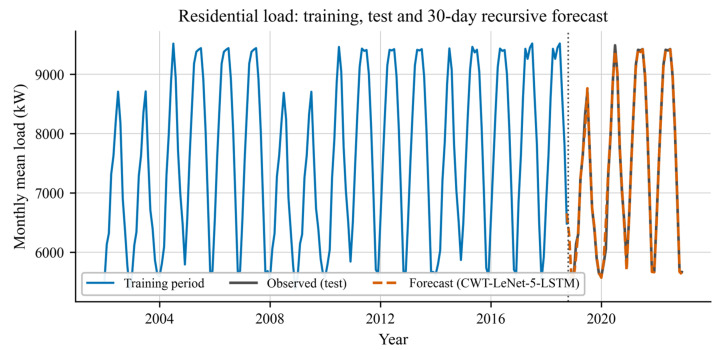
Annual range of electrical load consumption, training versus test versus prediction.

Figure 21 presents the performance for the out-of-sample forecasting and temporal partition obtained using the proposed CWT-LeNet-5-LSTM model.

The blue curve shows the training dataset for the period 2002–2018 while the gray line illustrates the obtained outcome of the test for the period 2018–2022 and both these curves are distinguished through a vertical dotted line. In the test period, the 30-day recursive forecast results appearing as dashed orange line are directly assessed against the actual data and these curves shpw good alignment.

### 5.9. Stratified Error Analysis

**Figure 22:**
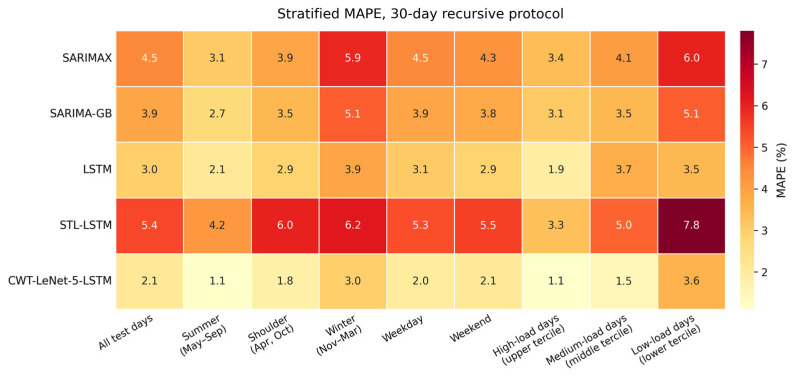
Stratified MAPE at lead 30.

The available series has daily rather than hourly resolutions; intraday peak and off-peak-hour errors cannot be evaluated. As a complementary operational analysis, test days are therefore divided into high-, medium- and low-load groups based on terciles of the observed load over the test span. Table 9 and Figure 22 additionally decompose the lead-30 error by season and by weekday against weekend. MAE and RMSE are reported alongside MAPE because a percentage error depends on the magnitude of the observed load in its denominator.

The stratification is more informative than the aggregate, and it does not point in the direction that might be assumed. Relative errors are lower during summer and on high-load days for every framework, along with the absolute errors. The proposed model shows an MAE of 96 kW for the summer against 183 kW in winter, and a high-load MAE of 96 kW against 209 kW on low-load days. The pattern is therefore not an artefact of the MAPE denominator; the frameworks are genuinely more accurate in kilowatts as well as in percentage terms, with higher accuracy during the high-demand summer months than during the low-demand winter months.

This is operationally favourable, because the largest absolute deviations do not coincide with the summer peak that governs reserve procurement. The difficult periods are instead the low-load winter days and the transitions into and out of the cooling season, where the series is proportionally more volatile. The weekday and weekend columns are indistinguishable for every framework, which is the expected consequence of the absence of a weekly cycle established in Section 4.3. The proposed framework records the lowest error in every stratum reported.

### 5.10. Statistical Significance

Table 10 reports Diebold–Mariano tests of every framework against CWT-LeNet-5-LSTM at lead 30, computed on squared-error loss differentials from identical forecast origins and identical targets, with the variance estimator truncated at the lead.

CWT-LeNet-5-LSTM has the lowest mean multi-step error, and its improvement over persistence, seasonal naive, SARIMAX, SARIMA-GB, and STL-LSTM is statistically significant at the 5% level. The difference relative to LSTM is not statistically significant on this test set and is not described. The conclusion supported by the evidence is that multi-scale hybridisation gives a consistent and statistically meaningful advantage over the linear and residual-corrected statistical frameworks under multi-step forecasting, not that it dominates every alternative under every protocol.

## 6. Discussion

### 6.1. Why Multi-Scale Hybridisation Helps, and Where It Does Not

The proposed architecture wins where the signal has energy at several time scales simultaneously. The scalogram makes short-lived excursions explicit as localized ridges at low scales while the annual envelope occupies the high scales, and the anisotropic pooling of Section 3.3.2 preserves the temporal index so that the recurrent stage can associate a ridge with the day on which it occurred. A plain recurrent network must infer that separation from raw amplitudes, and a linear seasonal model cannot represent it. The ablation of Section 5.7 shows that removing either the wavelet stage or the convolutional stage degrades the multi-step result, and replacing the anisotropic pooling with symmetric pooling degrades it further. It also explains why the margin widens with the lead: further ahead, a model must propagate its own predictions the more it depends on having represented the slow components correctly.

The architecture does not help, and is not worth its cost, at lead 1 when the previous day’s load is observed, and the lag-one autocorrelation is 0.978. The conditional mean is almost fully determined by a single lag, and a gradient-boosted residual learner with explicit lag features that exploits more directly than a wavelet convolution stack. Reporting only the one-step figure therefore risks attributing to the architecture an advantage that belongs to the protocol and, as Table 8 shows, can equally hide a genuine advantage that appears only further out.

### 6.2. Comparison with the State of the Art

Studies at the level of an aggregated distribution feeder with a multi-day lead typically report MAPE in the low single digits. Household-level short-term forecasting commonly reports double-digit MAPE because individual demand is far noisier than its aggregate. The tutorial review of Hong and Fan [60] and the Global Energy Forecasting Competition series [61] both emphasize this dependence. The 2.35 ± 0.28% obtained here at lead 30 on an aggregated residential feeder is consistent with that range. It should not be read as evidence of a generally superior method; for the reason given in Section 6.5, this particular series is unusually smooth, and its short-lead predictability is correspondingly high.

The comparison with CNN-LSTM hybrids reported in [24,25,26] and with the TFT and TiDE architecture evaluated at scale in [22] is instructive. Those studies consistently find that hybrid and attention-based models outperform classical statistical baselines, and the present results agree at multi-step leads. The fact that well-specified statistical models with good feature engineering remain highly competitive and are sometimes superior at short leads is precisely the pattern of Table 7. The contribution of this study is therefore not the claim that a deep hybrid is universally better, but the demonstration, using a utility record, that the answer depends on the lead, together with a protocol that makes that dependence visible and an ablation that identifies which components of the hybrid are responsible.

### 6.3. Physical Interpretation of the Load Pattern

The shape of the LESCO residential series reflects identifiable physical drivers, and these explain both the successes and the failures of the models.

The dominant annual cycle, with a July mean of roughly 9273 kW against a December mean of roughly 5669 kW, is thermostatically driven. Central Pakistan experiences summer temperatures that make air conditioning and evaporative cooling the largest single component of residential demand. Cooling load responds to temperature through a strongly non-linear threshold: below the comfort set-point, it is essentially zero; above it, it grows steeply. This is why the seasonal component is not sinusoidal and why four Fourier harmonics rather than one are required to represent it, and it is why the residual variance of the linear model concentrates in summer.

The near absence of a weekly cycle, at first sight surprising for an electricity series, is equally interpretable. Weekly rhythms in aggregate demand originate mainly in commercial and industrial activity. On a purely residential feeder, in a context where a substantial share of the population does not observe a standardized five-day working week, occupancy on Saturday differs little from occupancy on Wednesday, and cooling load follows occupancy. The 0.22% weekday spread measured in Section 4.3 is a property of the load rather than a deficiency of the data, and it is why the weekday and weekend strata of Section 5.9 are indistinguishable.

The wider context of residential demand in this region includes the prolonged supply shortfall and administrative load-shedding in the late 2000s. It also includes the rising adoption of rooftop PVs as tariffs increased and changes in daytime occupancy during 2020. These factors are documented in the energy policy literature and are the correct framework for interpreting residential demand in central Pakistan. The present record, however, contains no tariff, temperature or photovoltaic capacity covariate, and no customer-level export measurements, so their individual signatures cannot be separated from one another within this analysis. Attributing specific inter-annual movements to specific causes would require a record with those covariates attached, and no such attribution is made.

### 6.4. Methodological Outlook

Three directions follow from Section 2.1:

- First, structure encoding rather than structure learning: The exogenous Fourier block used here encodes the astronomical cycle. But the thermostatic response that generates the summer peak is a physical relation between ambient temperature and cooling demand. We advise encoding that relation as a constraint in the spirit of physics-informed learning [48] and of the model-integrated formulation of Huang et al. [49].
- Second, global-then-local estimation: The two-stage design of Zhu et al. [50], in which a global model trained across many units is adapted per unit from a small local sample, maps onto a distribution utility operating across hundreds of feeders and would also supply the per-feeder uncertainty quantification that a single point forecast lacks.
- Third, explicit adaptation to drift: Tariff changes, PV uptake and shifts in occupancy are textbook concept drift [52], and online or continual updating with drift detection is the natural response.

### 6.5. Limitations and Threats to Validity

The following limitations qualify the results and should be read alongside them:

- The series is unusually smooth for a load record, the lag-one autocorrelation is 0.978, and persistence alone achieves 2.61% MAPE at lead 1. Absolute error figures from this study should therefore not be compared directly with those from noisier feeders or from household-level data.
- The study is univariate in the endogenous variable; no weather, tariff or photovoltaic capacity covariate accompanies the record, although Section 6.3 argues that temperature is the proximate physical driver of the dominant seasonal component. The reported accuracy is what is achievable from calendar information and load history alone.
- Conventional consumers and prosumers are not separately identified in the record. The absence of customer-level PV capacity and export data prevents separate attribution of load changes to prosumer activity.
- Because the series is daily, intraday peak and off-peak-hour behaviour cannot be analysed; the load-band stratification of Section 5.9 is a day-level substitute, not an intraday analysis.
- Results are from a single feeder of a single utility. No claim of transfer to other feeders, utilities or countries is made or tested.
- Deep frameworks were trained under three random initialisations and are reported as the mean and standard deviation. Differences between frameworks smaller than that spread should not be interpreted. The Diebold–Mariano tests of Section 5.10 are the appropriate basis for judging which differences are meaningful. The architecture ablation of Section 5.7 was run with a single seed and indicates the direction of each effect rather than a precise magnitude.

## 7. Conclusions

This paper compared statistical, hybrid and deep learning frameworks—SARIMAX, SARIMA-GB, an LSTM network, STL decomposition with LSTM, and CWT-LeNet-5-LSTM—for forecasting residential electricity demand in the LESCO region of central Pakistan. All frameworks were fitted to the same 7670-point daily record spanning 1 January 2002 to 31 December 2022, under the same chronological partition and exogenous calendar block, and were evaluated under one recursive protocol at leads of 1, 7, 14 and 30 days against persistence and seasonal-naive references.

At lead 30, which corresponds to the monthly planning task the framework is intended for, CWT-LeNet-5-LSTM attained an MAPE of 2.35 ± 0.28% against 3.32% for the multivariate LSTM, 3.89% for SARIMA with gradient boosting, 4.47% for SARIMAX and 5.45% for STL with LSTM, with an MAE of 138 kW and an RMSE of 265 kW. Diebold–Mariano tests confirm that the advantage over the statistical frameworks is significant at the 5% level. At lead 1, the ranking changes, with the SARIMA-GB hybrid leading by 0.76%, because the previous day’s observed load dominates a one-step prediction on a series with lag-one autocorrelation of 0.978. The full range of leads is what makes this distinction visible, and the principal methodological recommendation of this study is that comparative load-forecasting work should state its protocol explicitly and report more than one lead.

The execution of such load estimation mechanisms is significant for precisely matching load demand and avoiding energy and fuel revenue losses in developing countries such as Pakistan. The residential segment is the largest power-consuming category in Pakistan, and the growth of rooftop solar PV is introducing variations in net demand that make accurate feeder-level forecasting more valuable rather than less.

Calendar variables are now supplied to every model as exogenous inputs, and a stratified error analysis across seasons, weekdays and weekends, and high-, medium- and low-load days is reported in Section 5.9. The limitations that remain are the smoothness of the series, the absence of weather and PV covariates, the daily resolution, the single-feeder scope and the variability across random initialisations. The inability to separate prosumer activity is more related to the data accumulation mechanism, and it can also be considered if the prosumer datasets are separately available.

## Figures and Tables

**Table 1:** Prominent neural networks for load and price forecasting.

Technique	Strength	Limitations	Computational Complexity	Ref.
ARIMA	High interpretability; effective for stationary series.	Limited to linear relationships; requires stationarity.	O(*n* log *n*).	[27]
CNN	Excellent feature extraction and pattern recognition.	Needs large datasets and high computational resources.	O(*n k*^2^ *m*) with layer and filter size.	[25,28]
RNN	Handles sequential data; maintains hidden state.	Vanishing gradients; difficult to train.	O(*n m*^2^) per time step.	[29,30]
DL	Feature layers learned automatically from data.	Slow convergence; low empirical coverage probability.	Architecture-dependent.	[31]
SVM	Handles high-dimensional data; robust on small datasets.	Costly for large datasets.	Depends on dataset size and kernel.	[31]
LSTM	Captures long-term dependencies; mitigates vanishing gradients.	Complex; longer training time.	O(*n m*^2^) per step with gating overhead.	[25,28]
SARIMAX	Extends SARIMA with exogenous variables; handles trend and seasonality.	Still linear; requires stationarity and careful variable selection.	O(*n k*) per iteration, *k* = number of parameters.	[32]

**Table 2:** Prominent ML and DL techniques hybridised with neural networks for load forecasting.

Technique	Strength	Limitations	Computational Complexity	Ref.
ANN-LSTM	Used for irregular datasets.	Training speed scales poorly with task complexity.	-	[33,34]
GHSA-FTS-LS-SVM	Improved computation accuracy and speed; higher forecasting precision.	Manual selection of parameters.	Dominated by RBF kernel parameter search.	[35]
SARIMA (gradient boosting)	SARIMA captures seasonality and linear components; boosting corrects the residual structure.	The residual learner can only correct structure the base model already represents; if the seasonal period of the base model is misspecified, the hybrid cannot recover the missing cycle.	O(*np* + *nP*) per likelihood evaluation.	[36]
LSTM with STL decomposition	STL isolates trend, seasonal and remainder components before modelling.	Decomposition may misallocate variance; pipeline more complex.	STL O(*n*); LSTM O(*n d h*^2^) per epoch.	[37]
CWT-LeNet-5-LSTM	CWT captures localised time–frequency patterns, LeNet-5 extracts spatial features and LSTM models long-range dependencies.	Increased training time and tuning difficulty; reduced interpretability; requires large training data.	Dominated by the deep learning stages.	[38]

**Table 3:** Specification of the analysed record.

Item	Value
Source	LESCO residential distribution feeder, central Pakistan
Start date	1 January 2002
End date	31 December 2022
Native temporal resolution	Daily (no aggregation is applied)
Number of observations	7670
Target variable	Residential_Plot
Quantity and unit	Daily aggregate residential feeder demand, kW
Mean/standard deviation	7546 kW/1417 kW
Minimum/maximum	5015 kW/9998 kW
Missing target values	0
Duplicate or missing dates	0; the index is a complete daily calendar
Outlier treatment	None applied; no physically impossible values present
Other columns in the file	Industry, Hospital, Park and Rescue feeders; excluded from this study
Exogenous measurements available	None; no weather, tariff or photovoltaic capacity data accompany the record
Prosumers separately identified	No; the series is aggregate feeder demand and may embed net-load effects

**Table 4:** Fitted exogenous coefficients of the SARIMAX (3, 1, 1) model.

Regressor	Coefficient (kW)	Std. Error	Z	*p*-Value
Annual sine, k = 1	361.6	84.1	4.30	<0.001
Annual cosine, k = 1	−1695.2	98.3	−17.24	<0.001
Annual sine, k = 2	240.0	47.0	5.10	<0.001
Annual cosine, k = 2	−31.4	45.7	−0.69	0.492
Annual sine, k = 3	−32.3	33.6	−0.96	0.336
Annual cosine, k = 3	3.4	31.0	0.11	0.913
Annual sine, k = 4	142.6	23.4	6.09	<0.001
Annual cosine, k = 4	14.8	23.7	0.62	0.532
Day-of-week sine	7.4	7.6	0.99	0.324
Day-of-week cosine	−4.7	5.4	−0.87	0.386
Weekend indicator	4.2	10.8	0.39	0.697
Linear trend (per year)	−6.5	383.5	−0.02	0.986

**Table 5:** Complete configuration of the five frameworks.

Component	Setting
Series	Daily residential feeder load, 7670 points, 1 January 2002 to 31 December 2022
Partition	Fit 5216/validation 920/test 1534 days, chronological
Scaling	z-score using fitting split statistics only
Exogenous block	14 variables: 4 annual Fourier harmonic pairs (8), day-of-week sine and cosine (2), month sine and cosine (2), weekend indicator (1), linear trend (1)
SARIMAX	Order (3, 1, 1), no seasonal ARMA polynomial, exogenous block as above; order selected by validation MAPE
Gradient boosting	HistGradientBoosting, squared error, depth 6, learning rate 0.05, 600 iterations with internal early stopping
STL	Period 365, robust Loess, fitted on the training span only, trend extrapolated linearly, seasonal component indexed by day of year
LSTM	4 layers × 50 units, dropout 0.2, input window 128 days × 15 channels (1 endogenous + 14 exogenous)
CWT	PyWavelets Morlet continuous wavelet (“morl”), 32 integer scales a = 1, 2,..., 32, magnitude retained, standardised on the fitting split
LeNet-5 stage	Conv2D 6 @ 5 × 5, stride 1, padding “same”, tanh; AvgPool 2 × 1, stride (2, 1); Conv2D 16 @ 5 × 5, stride 1, padding “same”, tanh; AvgPool 2 × 1, stride (2, 1)
Recurrent stage	LSTM 64 units, dropout 0.2, late fusion with the calendar vector, Dense 32 (ReLU), Dense 1
Trainable parameters	54,797 (proposed model); 74,651 (plain LSTM)
Optimiser	initial learning rate 1 × 10^−3^, batch size 64, gradient norm clipped at 1.0
Early stopping	Patience of 15 epochs on validation MSE, best weights restored; learning rate halved after 6 stagnant epochs; maximum 120 epochs
Random seeds	1, 7, 42 (3 independent runs per deep framework)
Software	PyTorch 2.5 (CPU), statsmodels 0.14, scikit-learn 1.7, PyWavelets 1.8

**Table 7:** Test set accuracy of all frameworks. Columns 2–5 are lead 1; columns 6–9 are lead 30.

Framework	MAE (kW)	RMSE (kW)	MAPE (%)	nRMSE (%)	MAE (kW)	RMSE (kW)	MAPE (%)	nRMSE (%)
Persistence	190	298	2.61	2.98	458	595	6.24	5.95
Seasonal naïve	640	975	8.82	9.75	642	976	8.85	9.76
SARIMAX	182	269	2.52	2.69	315	410	4.47	4.10
SARIMA-GB	53	86	0.76	0.86	275	371	3.89	3.71
LSTM	167	245	2.33	2.45	214	302	3.04	3.02
STL-LSTM	196	272	2.75	2.72	369	481	5.37	4.81
CWT-LeNet-5-LSTM	70	105	1.03	1.05	138	265	2.06	2.66

**Table 8:** MAPE (%) as a function of forecast lead. Deep frameworks are reported as mean ± standard deviation over 3 random initialisations.

Framework	Lead 1	Lead 7	Lead 14	Lead 30
Persistence	2.61	3.87	4.44	6.24
Seasonal naïve	8.82	8.83	8.87	8.85
SARIMAX	2.52	3.34	3.77	4.47
SARIMA-GB	0.76	2.27	2.91	3.89
LSTM	2.54 ± 0.19	2.83 ± 0.18	2.98 ± 0.22	3.32 ± 0.37
STL-LSTM	2.86 ± 0.10	4.01 ± 0.19	4.64 ± 0.20	5.45 ± 0.18
CWT-LeNet-5-LSTM	1.20 ± 0.17	1.46 ± 0.28	1.77 ± 0.30	2.35 ± 0.28

**Table 9:** Stratified error at lead 30, with each cell reporting MAE (kW)/RMSE (kW)/MAPE (%).

Stratum	*N*	SARIMAX	SARIMA-GB	LSTM	STL-LSTM	CWT-LeNet-5-LSTM
All test days	1530	315/410/4.47	275/371/3.89	214/302/3.04	369/481/5.37	138/265/2.06
Summer (May–Sep)	612	265/371/3.10	232/329/2.71	178/267/2.14	343/468/4.19	96/214/1.14
Shoulder (Apr, Oct)	256	290/364/3.94	259/337/3.51	217/286/2.94	427/525/6.04	122/195/1.75
Winter (Nov–Mar)	662	371/457/5.95	320/416/5.13	248/337/3.91	371/475/6.20	183/326/3.03
Weekdays	1092	318/410/4.54	276/369/3.93	218/298/3.09	366/476/5.33	137/267/2.05
Weekends	438	307/409/4.31	270/375/3.79	207/311/2.93	376/494/5.47	140/261/2.10
High-load days (upper tercile)	510	307/420/3.38	279/393/3.06	176/266/1.94	298/393/3.31	96/212/1.07
Medium-load days (middle tercile)	510	294/401/4.06	251/352/3.47	265/364/3.70	365/478/5.03	109/162/1.51
Low-load days (lower tercile)	510	344/407/5.98	295/365/5.13	203/265/3.48	443/558/7.76	209/375/3.61

**Table 10:** Diebold–Mariano tests against CWT-LeNet-5-LSTM at lead 30. A positive statistic favours CWT-LeNet-5-LSTM.

Comparison	Lead	DM Statistic	*p*-Value	Interpretation
CWT-LeNet-5-LSTM vs. persistence	30	4.88	<0.001	Significant at 5%
CWT-LeNet-5-LSTM vs. seasonal naïve	30	3.28	0.00105	Significant at 5%
CWT-LeNet-5-LSTM vs. SARIMAX	30	3.00	0.00271	Significant at 5%
CWT-LeNet-5-LSTM vs. SARIMA-GB	30	2.11	0.0346	Significant at 5%
CWT-LeNet-5-LSTM vs. LSTM	30	0.72	0.469	Not significant
CWT-LeNet-5-LSTM vs. STL-LSTM	30	4.98	<0.001	Significant at 5%

## Data Availability

The datasets can be provided for research purposes only and through email communication with primary author.
